# Factors Affecting Patients’ Use of Electronic Personal Health Records in England: Cross-Sectional Study

**DOI:** 10.2196/12373

**Published:** 2019-07-31

**Authors:** Alaa Abd-Alrazaq, Bridgette M Bewick, Tracey Farragher, Peter Gardner

**Affiliations:** 1 Leeds Institute of Health Sciences School of Medicine University of Leeds London United Kingdom; 2 Division of Information and Computing Technology College of Science and Engineering Hamad Bin Khalifa University Doha Qatar; 3 School of Psychology University of Leeds Leeds United Kingdom

**Keywords:** health records, personal, patient portal, electronic personal health records, technology acceptance, technology adoption, intention, unified theory of acceptance and use of technology, structural equation modelling

## Abstract

**Background:**

Electronic personal health records (ePHRs) are secure Web-based tools that enable individuals to access, manage, and share their medical records. England recently introduced a nationwide ePHR called Patient Online. As with ePHRs in other countries, adoption rates of Patient Online remain low. Understanding factors affecting patients’ ePHR use is important to increase adoption rates and improve the implementation success of ePHRs.

**Objective:**

This study aimed to examine factors associated with patients’ use of ePHRs in England.

**Methods:**

The unified theory of acceptance and use of technology was adapted to the use of ePHRs. To empirically examine the adapted model, a cross-sectional survey of a convenience sample was carried out in 4 general practices in West Yorkshire, England. Factors associated with the use of ePHRs were explored using structural equation modeling.

**Results:**

Of 800 eligible patients invited to take part in the survey, 624 (78.0%) returned a valid questionnaire. Behavioral intention (BI) was significantly influenced by performance expectancy (PE; beta=.57, *P*<.001), effort expectancy (EE; beta=.16, *P*<.001), and perceived privacy and security (PPS; beta=.24, *P*<.001). The path from social influence to BI was not significant (beta=.03, *P*=.18). Facilitating conditions (FC) and BI significantly influenced use behavior (UB; beta=.25, *P*<.001 and beta=.53, *P*<.001, respectively). PE significantly mediated the effect of EE and PPS on BI (beta=.19, *P*<.001 and beta=.28, *P*=.001, respectively). Age significantly moderated 3 paths: PE→BI, EE→BI, and FC→UB. Sex significantly moderated only the relationship between PE and BI. A total of 2 paths were significantly moderated by education and internet access: EE→BI and FC→UB. Income moderated the relationship between FC and UB. The adapted model accounted for 51% of the variance in PE, 76% of the variance in BI, and 48% of the variance in UB.

**Conclusions:**

This study identified the main factors that affect patients’ use of ePHRs in England, which should be taken into account for the successful implementation of these systems. For example, developers of ePHRs should involve patients in the process of designing the system to consider functions and features that fit patients’ preferences and skills to ensure systems are useful and easy to use. The proposed model accounted for 48% of the variance in UB, indicating the existence of other, as yet unidentified, factors that influence the adoption of ePHRs. Future studies should confirm the effect of the factors included in this model and identify additional factors.

## Introduction

### Background

Electronic personal health records (ePHRs) refer to secure Web-based tools that enable individuals to access and manage their medical records and share them with trusted others [1]. More advanced ePHRs provide additional functionalities, such as scheduling appointments, requesting prescription refills, messaging providers, requesting referrals, and educational tools [2-4]. Benefits of using ePHRs include the following: enhancing patient empowerment [5,6], improving patient self-management and medication adherence [7,8], enhancing the relationships and communications between patients and health care providers [9,10], enabling patients to easily access health services [11,12], avoiding duplicated tests [9,11], and reducing adverse drug interactions and allergies [9,11,13].

In 2015, the National Health Service in England launched a program called Patient Online, which requires general practices (GPs) to provide patients with Web-based services, such as booking appointments, requesting prescription refills, and viewing summary information from GP records [14,15]. GPs use one of the following systems to provide their patients with the abovementioned services: SystemOnline, Patient Access, Patient Services, The Waiting Room, Engage Consult, and Evergreen Life or i-Patient [14].

### Research Problem and Aim

The overall adoption rate of Patient Online was 18.9% in April 2017 and reached 24.4% in April 2018 [16], and so adoption remains low. Identifying and understanding factors that affect patients’ use of ePHRs is crucial to develop interventions to increase patients’ adoption and improve the implementation success of ePHRs [17-22]. According to a systematic review conducted by Abd-alrazaq and colleagues [23], there are no published studies on factors affecting patients’ use of ePHRs in England. Although many studies have been conducted in other countries, they have several shortcomings, namely, (1) few studies were theory-based research [21,24-27], (2) many studies focused on factors that affect patients’ intention to use ePHRs instead of actual use [28-32], (3) many studies have assessed the factors that affect self-reported use rather than actual use [27,32-35], (4) almost all studies examined independent and dependent variables at one point in time using the same data collection instrument, so being at risk of common method bias [25,32,36], and (5) almost all studies did not differentiate between factors affecting initial use and continuing use of ePHRs.

This study aimed to examine factors associated with patients’ adoption of ePHRs (Patient Online) in England. As 76% of patients in England have never used Patient Online [16], the study focused on factors associated with patients’ initial use of ePHRs. Therefore, it was more appropriate to investigate the factors that make nonusers become users (ie, initial use stage).

## Methods

### Theoretical Foundation

In total, 12 theories and models originated from various disciplines, such as psychology, sociology, and information systems, were reviewed to select the appropriate one for our study. Selection of the appropriate theory was based on predefined 6 criteria. Although 2 criteria were related to the applicability of the theory on the phenomena of interest (ie, population and type of behavior), the remaining 4 were related to goodness of the theory (ie, logical consistency, explanatory power, falsifiability, and parsimony). The unified theory of acceptance and use of technology (UTAUT) was the only theory that met all those criteria. Therefore, this study chose UTAUT as a theoretical lens to examine factors associated with patients’ use of ePHRs. More details about how the theories met or did not meet each criterion are explained in Multimedia Appendix 1.

According to UTAUT, behavioral intention (BI) is affected directly by performance expectancy (PE), effort expectancy (EE), and social influence (SI) [37]. Both BI and facilitating conditions (FC) are hypothesized to affect use behavior (UB) directly [37]. UTAUT also proposes that most of these relationships are moderated by age, sex, experience, and voluntariness [37].

In this study, the adoption of ePHRs is not compulsory. The UTAUT construct of voluntariness is only applicable in nonvoluntary contexts [38]. Thus, for this study, the moderator *voluntariness* was dropped from the model. This study focused on the factors that explained how nonusers become users of ePHRs (ie, preusage stage); the sample comprised only nonusers of ePHRs (ie, having no experience). For that reason, the moderator *experience* was also removed from the model.

**Figure 1:**
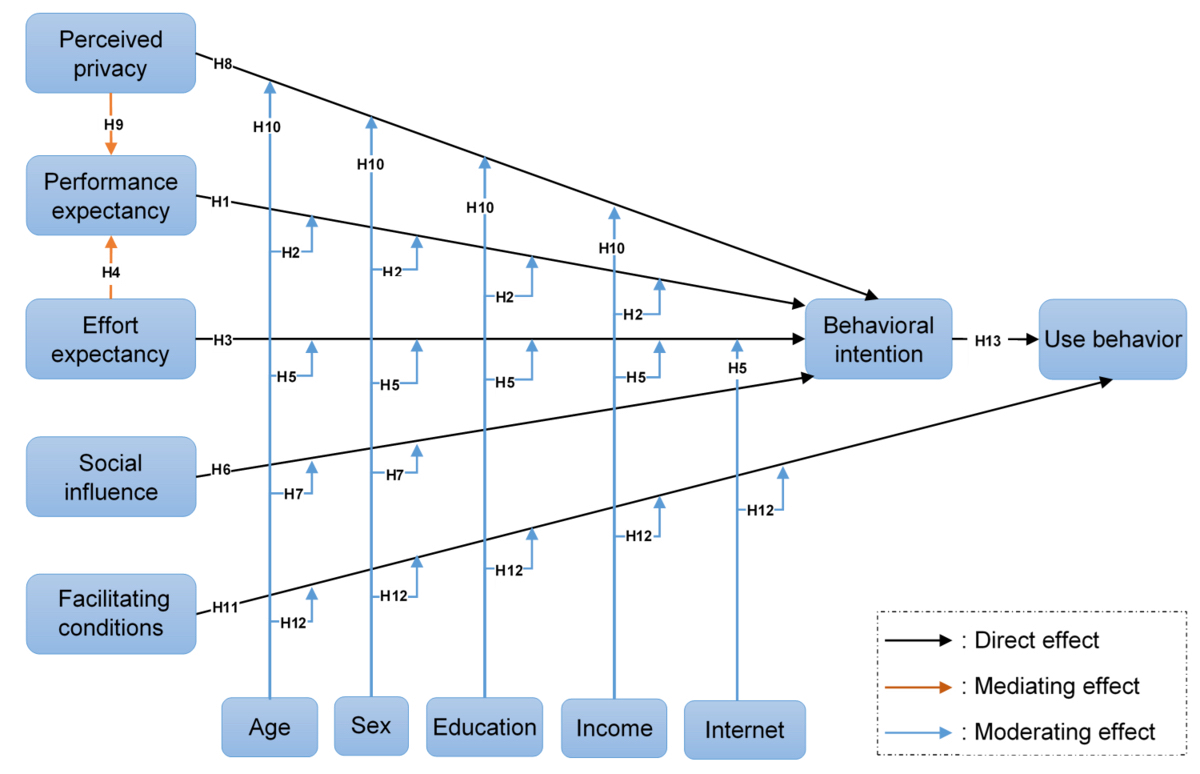
The proposed model.

A review of the literature identified a consensus on the influential effect of the following factors on ePHRs adoption: PPS [26,39-48], internet access [11,28,39,49-53], income [26,28,39,49,51,54-58], and education level [26,28,39,44,49,51,56,59-63]. These 4 factors were not part of UTAUT but were included in our adapted model to make it more appropriate for the context of ePHRs adoption. Although PPS was proposed as an independent variable, the remaining 3 factors were hypothesized as a moderator. The research hypotheses and the proposed model are presented in Table 1 and Figure 1, respectively. Multimedia Appendix 2 shows the conceptual definitions of the constructs in the proposed model. Multimedia Appendix 3 shows the theoretical foundations for the new proposed relationships that were added to the UTAUT model.

**Table 1:** The research hypotheses.

H^a^ number	Hypothesis
H1	PE^b^ positively influences patients’ intention to use Patient Online.
H2	Age, sex, education, and income moderate the positive relationship between PE and patients’ intention to use Patient Online, such that the influence is stronger for younger males with lower level of education and higher income.
H3	EE^c^ positively influences patients’ intention to use Patient Online.
H4	PE positively mediates the positive relationship between EE and BI^d^.
H5	Age, sex, education, income, and internet access moderate the positive relationship between EE and patients’ intention to use Patient Online, such that the influence is stronger for older females with lower level of education and income and without internet access.
H6	SI^e^ positively influences patients’ intention to use Patient Online.
H7	Age and sex moderate the positive relationship between SI and patients’ intention to use Patient Online, such that the influence is stronger for older females.
H8	PPS^f^ positively influences patients’ intention to use Patient Online.
H9	PE positively mediates the positive relationship between PPS and BI.
H10	Age, sex, education, and income moderate the positive relationship between PPS and patients’ intention to use Patient Online, such that the influence is stronger for older females with higher level of education and lower income.
H11	FC^g^ positively influences patients’ use of Patient Online.
H12	Age, sex, education, income, and internet access moderate the positive relationship between FC and UB^h^, such that the influence is stronger for older females with a lower level of education and income and without internet access.
H13	BI positively influences patients’ use of Patient Online.

^a^H: hypothesis.

^b^PE: performance expectancy.

^c^EE: effort expectancy.

^d^BI: behavioral intention.

^e^SI: social influence.

^f^PPS: perceived privacy and security.

^g^FC: facilitating conditions.

^h^UB: use behavior.

### Study Design and Setting

The proposed model was examined empirically using data from a cross-sectional survey. The survey was conducted at 4 West Yorkshire (England) GPs, 3 practices in Bradford and 1 in Leeds. More details about these practices are shown in Multimedia Appendix 4. Health Research Authority approval for this study was granted before starting data collection (REC reference: 17/SC/0323).

### Measurement

Self-administrated questionnaires were used to measure all variables proposed in the model except UB. UB was measured objectively using system logs that recorded the use of PatientOnline. Questionnaires included 29 well-validated items adopted from previous studies (Multimedia Appendix 5). An introduction about Patient Online was included at the top of the questionnaire to ensure all participants had the knowledge necessary to answer questions about Patient Online. The questionnaire was validated by sending it to a panel of experts to assess the face validity and content validity of the questions. After modifying the questionnaire according to experts’ recommendations, it was pilot tested by sending it via email to 37 patients (members of patient and carer community) who were asked to fill in the questionnaire and answer questions regarding clarity or ambiguity of questions, clarity of instructions to answer questions, difficulty to answer questions, time needed to complete the questionnaire, clarity and attractiveness of the layout, missing of important topics, and sequence of questions. A few issues were reported by experts and patients, and the questionnaire was modified accordingly (Multimedia Appendix 6). System log data of the number of times that each participant logged into the system during 6 months after completing the questionnaire were the objective measure of use.

### Recruitment

We recruited a convenience sample of patients from August 21, 2017, to September 26, 2017. Patients were eligible to participate if they (1) lived in England and were registered at 1 of the 4 GPs, (2) were aged 18 years or older, and (3) had not used Patient Online before (nonusers). The researcher distributed the questionnaire to eligible participants visiting 1 of the 4 GPs. After 6 months from the completion of the questionnaire, data from the system log were extracted to ascertain participants’ use of Patient Online.

### Statistical Analysis

Structural equation modeling (SEM) was used to test the theoretical model and hypotheses. Specifically, the measurement model was examined in terms of 3 aspects: model fit, construct reliability, and construct validity [64,65]. After ensuring the validity of the measurement model, the structural model was tested in terms of 3 aspects: model fit, predictive power, and strength of relationships [65-67]. The strength of relationships was examined using different methods depending on the type of the proposed effect. Specifically, direct effects were assessed by checking path coefficients [68]. Mediating effects were examined by assessing the indirect effect using bootstrapping. The moderating effect for the metric moderator (ie, age) was examined using the interaction effect method [64,69]. The moderating effects for nonmetric moderators were tested using multigroup SEM [64,69,70]. Analysis of moment structures (version 24; IBM SPSS) software was used for conducting all abovementioned analyses.

## Results

### Participants’ Characteristics

Of the 800 eligible patients invited to take part in the survey, 624 participants returned a completed questionnaire giving a response rate of 78%. The mean age of participants was 44.2 years. The majority of participants were white (79.8%, 498/624) and had internet access (84.6%, 528/624; Table 2). Differences between participants and nonparticipants in terms of age, sex, and ethnicity were not significant (*P*=.21, *P*=.06, and *P*=.64, respectively). It was, therefore, concluded that the risk of nonresponse bias was minimal.

**Table 2:** Participants’ characteristics (n=624).

Variables		Respondents, n (%)
**Age (years)^a^**		
	18-24	107 (17.1)
	25-34	148 (23.7)
	35-44	116 (18.6)
	45-54	98 (15.7)
	55-64	65 (10.4)
	65-74	46 (7.4)
	75 and older	44 (7.1)
**Sex**		
	Male	293 (46.9)
	Female	331 (53.1)
**Ethnicity**		
	White	498 (79.8)
	Asian	73 (11.7)
	Black	20 (3.2)
	Mixed	26 (4.1)
	Others	7 (1.2)
**Income (£)**		
	<20,000	284 (45.5)
	20,000-29,999	80 (12.8)
	30,000-39,999	65 (10.4)
	40,000-49,999	43 (7.0)
	50,000-59,999	26 (4.2)
	≥60,000	12 (1.9)
	Prefer not to say	114 (18.2)
**Education**		
	Up to secondary school	69 (11.1)
	Secondary school	147 (23.6)
	College/diploma	165 (26.4)
	Bachelor’s degree	174 (27.9)
	Master’s degree	47 (7.5)
	Doctoral degree	22 (3.5)
**Internet access**		
	Yes	528 (84.6)
	No	96 (15.4)

^a^Mean 44.2 (SD 18.9).

### Measurement Model

#### Model Fit

All fit indices indicated a good fit of the initial model except the following 3 indices: goodness-of-fit index (GFI, 0.923), root mean square error of approximation (RMSEA, 0.053), and standardized root mean square residual (SRMR, 0.057; Table 3). The following 4 items were identified as a source of the poor fit of the measurement model as their factor loading was less than 0.70: FC 4, FC 5, PPS 3, and PPS 5. After deleting these 4 items from the model, all fit indices of the modified model improved and existed within their acceptable levels, indicating a good fit (Table 3).

**Table 3:** Results of fit indices of the initial and modified measurement model.

Fit indices	Cutoff point	Initial measurement model	Modified measurement model
Relative chi-square (*df*)	1-3	2.8 (215)	1.4 (137)
GFI^a^	≥0.95	0.923	0.969
AGFI^b^	≥0.90	0.902	0.957
RMSEA^c^	<0.05	0.053	0.026
PCLOSE^d^	≥0.05	0.194	1.000
SRMR^e^	≤0.05	0.057	0.017
NFI^f^	≥0.95	0.964	0.988
CFI^g^	≥0.95	0.977	0.995
TLI^h^	≥0.95	0.972	0.996

^a^GFI: goodness-of-fit index.

^b^AGFI: adjusted goodness-of-fit index.

^c^RMSEA: root mean square error of approximation.

^d^PCLOSE: p of close fit.

^e^SRMR: standardized root mean square residual.

^f^NFI: normed fit index.

^g^CFI: comparative fit index.

^h^TLI: Tucker-Lewis index.

#### Construct Reliability

Results for the modified model of Cronbach alpha, composite reliability, and average variance extracted (AVE) for each construct were within their cutoff of ≥.70, ≥0.70, and ≥0.50, respectively (Multimedia Appendix 7). This indicates that the measurement items are consistent and reproducible in measuring what it is assumed to measure.

#### Construct Validity

The values of factor loading and AVE for all items considerably exceeded the thresholds of 0.70 and 0.50, respectively (Multimedia Appendix 8). These results indicate that items had good convergent validity. Similarly, items showed good discriminant validity according to 3 measures. Specifically, intercorrelation coefficients are located within the acceptable ranges (<0.85; Multimedia Appendix 9). With regard to the second measure, each value of square root of AVE for a construct (values on the diagonal) was higher than all intercorrelation coefficients between that construct and each other construct (off-diagonal values; Multimedia Appendix 9). With regard to the third measure, the loading of each item on its construct was higher than cross-loadings in rows and columns (Multimedia Appendix 10).

### Structural Model

#### Model Fit and Predictive Power

**Figure 2:**
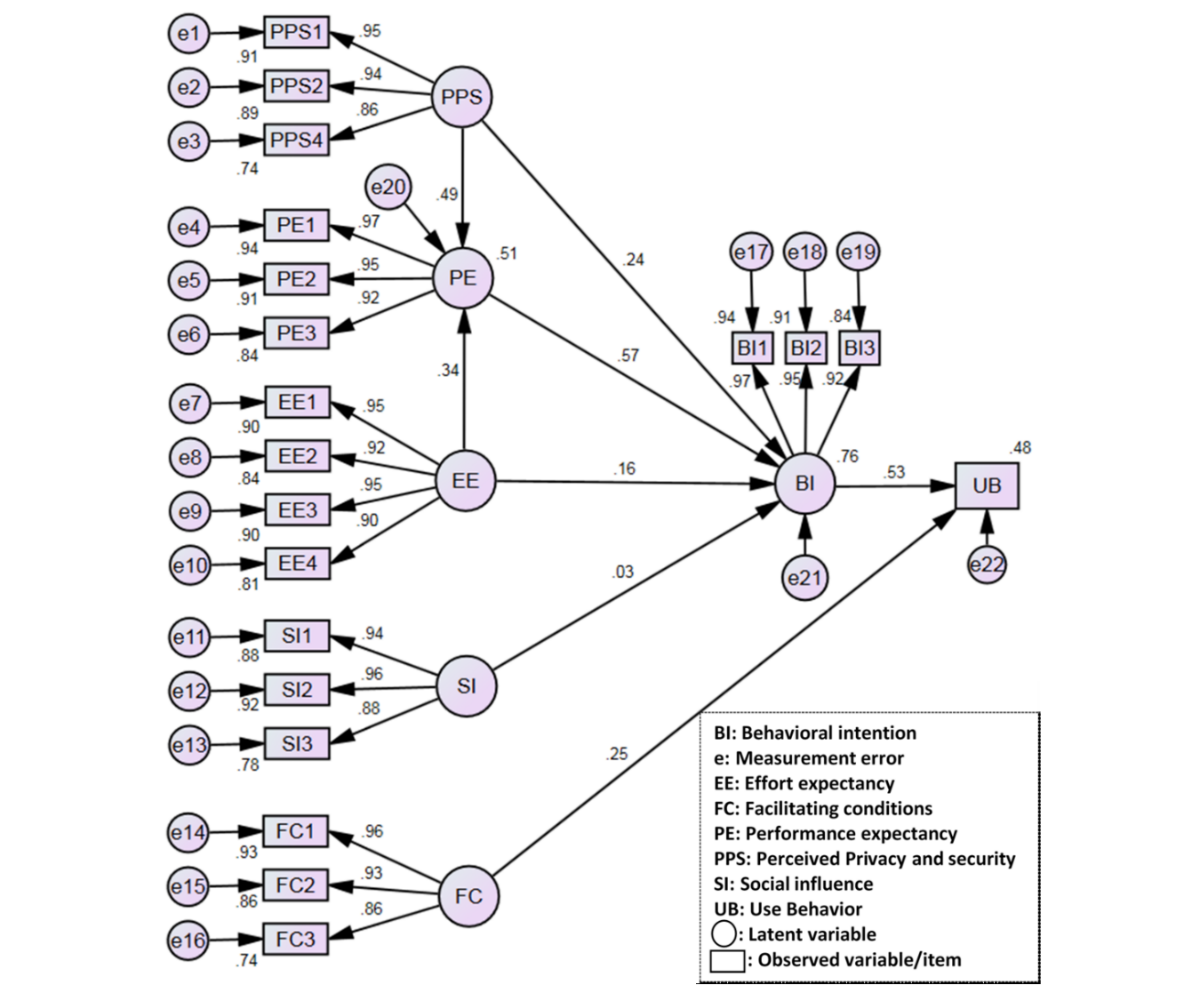
Structural model estimates.

All fit indices of the structural model indices were within their cutoff levels, indicating a good model fit (Table 4). The structural model accounted for 51% of the variance in PE, 76% of the variance in BI, and 48% of the variance in UB (Figure 2).

**Table 4:** Results of fit indices of the structural model.

Fit indices	Cutoff point	Fitness of the structural model
Relative chi-square (*df*)	1-3	1.6 (157)
GFI^a^	≥0.95	0.962
AGFI^b^	≥0.90	0.949
RMSEA^c^	<0.05	0.032
PCLOSE^d^	≥0.05	1.000
SRMR^e^	≤0.05	0.036
NFI^f^	≥0.95	0.984
CFI^g^	≥0.95	0.993
TLI^h^	≥0.95	0.992

^a^GFI: goodness-of-fit index.

^b^AGFI: adjusted goodness-of-fit index.

^c^RMSEA: root mean square error of approximation.

^d^PCLOSE: p of close fit.

^e^SRMR: standardized root mean square residual.

^f^NFI: normed fit index.

^g^CFI: comparative fit index.

^h^TLI: Tucker-Lewis index.

#### Strength of Relationships

Of the direct effects, BI was associated with PE (beta=.57), EE (beta=.16), and PPS (beta=.24; Table 5). The path from SI to BI was not significant (beta=.03, *P*=.18). Both FC and BI were significantly associated with UB (beta=.25 and beta=.53, respectively). Therefore, the following hypotheses were supported: H1, H3, H8, H11, and H13 (Table 5).

With regard to mediating effects, results of bootstrapping indicate that PE mediated significantly the effect of EE and PPS on BI (beta=.20 and beta=.28, respectively; Table 6). Accordingly, H4 and H9 were supported in this research.

With regard to moderating effects, age moderated significantly 3 paths: PE→BI (beta=−.10), EE→BI (beta=.06), and FC→UB (beta=.16; Table 7). Sex moderated significantly only the relationship between PE and BI (*P*=.009; Table 8). In relation to moderating effect of education, the relationship between FC and UB was statistically stronger for *secondary school or lower* group than college group (beta=.39 vs beta=.30, *P*=.003; Table 9) and than *bachelor or higher* group (beta=.39 vs beta=.21; Table 10). The path from EE to BI was statistically weaker for *bachelor or higher* group than *secondary school or lower* group (beta=.01 vs beta=.14; Table 10) and than college group (beta=.01 vs beta=.13; Table 11). As shown in Table 12-14, the relationship between FC and UB was statistically stronger for patients with low income (beta=.43) than patients with moderate or high income (beta=.25 and beta=.10, respectively). Internet access moderated significantly 2 paths EE→BI (*P*=.01) and FC→UB (*P*<.001; Table 15). Accordingly, H10 was rejected, whereas the following hypotheses were partially supported: H2, H5, H7, and H12.

**Table 5:** Results of direct effects.

H^a^	Path	SE (beta)	95% CI	*P* value	Supported?
H1	PE^b^^→^BI^c^	.57	0.51 to 0.64	<.001	Yes
H3	EE^d^→BI	.16	0.11 to 0.21	<.001	Yes
H6	SI^e^→BI	.03	−0.03 to 0.10	.18	No
H8	PPS^f^→BI	.24	0.18 to 0.29	<.001	Yes
H11	FC^g^→UB^h^	.25	0.20 to 0.30	<.001	Yes
H13	BI →UB	.53	0.48 to 0.58	<.001	Yes

^a^H: hypothesis.

^b^PE: performance expectancy.

^c^BI: behavioral intention.

^d^EE: effort expectancy.

^e^SI: social influence.

^f^PPS: perceived privacy and security.

^g^FC: facilitating conditions.

^h^UB: use behavior.

**Table 6:** Results of mediating effects.

H^a^	Indirect effect	Estimate (beta)	95% CI	*P* value	Supported?
H4	EE^b^→PE^c^→BI^d^	.20	0.15-0.25	<.001	Yes
H9	PPS^e^→PE→BI	.28	0.23-0.33	<.001	Yes

^a^H: hypothesis.

^b^EE: effort expectancy.

^c^PE: performance expectancy.

^d^BI: behavioral intention.

^e^PPS: perceived privacy and security.

**Table 7:** Results of moderating effect of age.

Interaction effect	Standardized estimate (beta)	*P* value
PE^a^×Age→BI^b^	−.10	<.001
EE^c^×Age→BI	.06	.03
SI^d^×Age→BI	.01	.06
PPS^e^×Age→BI	−.03	.22
FC^f^×Age→UB^g^	.16	<.001

^a^PE: performance expectancy.

^b^BI: behavioral intention.

^c^EE: effort expectancy.

^d^SI: social influence.

^e^PPS: perceived privacy and security.

^f^FC: facilitating conditions.

^g^UB: use behavior.

**Table 8:** Results of moderating effect of sex.

Hypothesized path	SE (beta)		*P* value		Chi-square difference test, *P* value
	Male	Female	Male	Female	
PE^a^→BI^b^	.59	.51	<.001	<.001	.01
EE^c^→BI	.17	.19	<.001	<.001	.32
SI^d^→BI	−.03	.06	.53	.06	.07
PPS^e^→UB	.27	.20	<.001	<.001	.65
FC^f^→UB^g^	.35	.28	<.001	<.001	.32

^a^PE: performance expectancy.

^b^BI: behavioral intention.

^c^EE: effort expectancy.

^d^SI: social influence.

^e^PPS: perceived privacy and security.

^f^FC: facilitating conditions.

^g^UB: use behavior.

**Table 9:** Results of moderating effect of education level (secondary school versus college/diploma).

Hypothesized path	Secondary school or lower		College/diploma		Chi-square difference test, *P* value
	SE (beta)	*P* value	SE (beta)	*P* value	
PE^a^→BI^b^	.57	<.001	.62	<.001	.38
EE^c^→BI	.14	.02	.13	.003	.38
PPS^d^→BI	.17	.005	.29	<.001	.22
FC^e^→UB^f^	.39	<.001	.30	<.001	.003

^a^PE: performance expectancy.

^b^BI: behavioral intention.

^c^EE: effort expectancy.

^d^PPS: perceived privacy and security.

^e^FC: facilitating conditions.

^f^UB: use behavior.

**Table 10:** Results of moderating effect of education level (secondary school versus bachelor or higher).

Hypothesized path	Secondary school or lower		Bachelor or higher		Chi-square difference test, *P* value
	SE (beta)	*P* value	SE (beta)	*P* value	
PE^a^→BI^b^	.57	<.001	.57	<.001	.50
EE^c^→BI	.14	.02	.01	.16	.03
PPS^d^→BI	.17	.005	.24	<.001	.14
FC^e^→UB^f^	.39	<.001	.21	<.001	.02

^a^PE: performance expectancy.

^b^BI: behavioral intention.

^c^EE: effort expectancy.

^d^PPS: perceived privacy and security.

^e^FC: facilitating conditions.

^f^UB: use behavior.

**Table 11:** Results of moderating effect of education level (college/diploma versus bachelor or higher).

Hypothesized path	College/diploma		Bachelor or higher		Chi-square difference test, *P* value
	SE (beta)	*P* value	SE (beta)	*P* value	
PE^a^→BI^b^	.62	<.001	.57	<.001	.08
EE^c^→BI	.13	.003	.01	.16	.008
PPS^d^→BI	.29	<.001	.24	<.001	.54
FC^e^→UB^f^	.30	<.001	.21	<.001	.23

^a^PE: performance expectancy.

^b^BI: behavioral intention.

^c^EE: effort expectancy.

^d^PPS: perceived privacy and security.

^e^FC: facilitating conditions.

^f^UB: use behavior.

**Table 12:** Results of moderating effect of income (low income versus middle income).

Hypothesized path	Low income^a^		Middle income^b^		Chi-square difference test, *P* value
	SE (beta)	*P* value	SE (beta)	*P* value	
PE^c^→BI^d^	.54	<.001	.52	<.001	.43
EE^e^→BI	.14	<.001	.22	<.001	.39
PPS^f^→BI	.26	<.001	.28	<.001	.99
FC^g^→UB^h^	.43	<.001	.25	<.001	.048

^a^Low income: >£20,000.

^b^Medium income: £20,000-39,999.

^c^PE: performance expectancy.

^d^BI: behavioral intention.

^e^EE: effort expectancy.

^f^PPS: perceived privacy and security.

^g^FC: facilitating conditions.

^h^UB: use behavior.

**Table 13:** Results of moderating effect of income (low income versus high income).

Hypothesized path	Low income^a^		High income^b^		Chi-square difference test, *P* value
	SE (beta)	*P* value	SE (beta)	*P* value	
PE^c^→BI^d^	.54	<.001	.68	<.001	.09
EE^e^→BI	.14	<.001	.12	.048	.67
PPS^f^→BI	.26	<.001	.25	<.001	.87
FC^g^→UB^h^	.43	<.001	.10	.03	.03

^a^Low income: >£20,000.

^b^High income: ≥£40,000.

^c^PE: performance expectancy.

^d^BI: behavioral intention.

^e^EE: effort expectancy.

^f^PPS: perceived privacy and security.

^g^FC: facilitating conditions.

^h^UB: use behavior.

**Table 14:** Results of moderating effect of income (middle income versus high income).

Hypothesized path	Middle income^a^		High income^b^		Chi-square difference test, *P* value
	SE (beta)	*P* value	SE (beta)	*P* value	
PE^c^→BI^d^	.52	<.001	.68	<.001	.06
EE^e^→BI	.22	<.001	.12	.048	.27
PPS^f^→BI	.28	<.001	.25	<.001	.88
FC^g^→UB^h^	.25	<.001	.10	.03	.02

^a^Medium income: £20,000-39,999.

^b^High income: ≥£40,000.

^c^PE: performance expectancy.

^d^BI: behavioral intention.

^e^EE: effort expectancy.

^f^PPS: perceived privacy and security.

^g^FC: facilitating conditions.

^h^UB: use behavior.

**Table 15:** Results of the moderating effect of internet access.

Hypothesized path	SE (beta)		*P* value		Chi-square difference test, *P* value
	Internet access	No internet access	Internet access	No internet access	
EE^a^→BI^b^	.12	.28	<.001	.005	.01
FC^c^→UB^d^	.18	.44	<.001	<.001	<.001

^a^EE: effort expectancy.

^b^BI: behavioral intention.

^c^FC: facilitating conditions.

^d^UB: use behavior.

## Discussion

### Principal Findings

This study found that PE was positively associated with BI. This means that patients are more likely to intend to use Patient Online when they perceive it to be very useful and advantageous. This relationship is consistent with other studies investigating the uptake of ePHRs [18,26,71,72-75]. Our results suggest that this relationship is stronger for younger males, meaning that younger males who perceive the system as more useful are more likely to intend to use it. This study is one of the very few studies that successfully assessed the moderating effect of age and sex in explaining the use of ePHRs. A similar moderating effect has been demonstrated for the use of consumer health information technologies (CHITs) [76,77].

These results showed that EE was positively associated with BI, that is, patients are more likely to intend to use Patient Online when they perceive it as an easy-to-use system. This finding is consistent with studies investigating the use of ePHRs outside of England [26,72,78-80]. This study showed that the relationship between EE and BI was mediated by PE. So, patients who perceive Patient Online as easy to use are more likely to perceive it as a useful system, thereby, they are more likely to intend to use it. This finding is in line with findings of 2 CHIT studies [22,81]. Furthermore, our results showed that the relationship between EE and BI was stronger among older patients with lower level of education and without internet access. The moderating effect of age has also been found in studies investigating the use of CHITs [76]. Ours is the first study to examine the moderating effect of education and internet access to explain the use of ePHRs or CHITs.

We found that SI and BI were not statistically associated. This means that opinions and beliefs of people who are important to the patient do not affect their intention to utilize ePHRs. This nonsignificant relationship could be attributed to the use of Patient Online being voluntary. The literature suggests that the effect of SI is significant only in contexts where using the technology is mandatory [37,82-84]. The presence of PE in a model may weaken the direct effect of SI on BI [85,86] as SI affects BI indirectly through PE [22,84]. The nonsignificant effect of SI may also be attributed to the fact that the questionnaire measures perceptions of SI rather than actual SI.

This study demonstrated that PPS was positively associated with BI, that is, patients are more likely to intend to use Patient Online when they perceive that it is secure and will maintain their privacy. This relationship is documented elsewhere in the literature [30,45,46,48,87,88]. This study showed that the relationship between PPS and BI was mediated by PE, that is, patients who perceive Patient Online to be secure and able to maintain their privacy are more likely to perceive it as a useful system and, therefore, are more likely to intend to use it. Although several studies in the context of ePHRs and CHITs examined the direct influence of PPS on PE and on BI, no previous study has tested the indirect effect of PPS on BI through PE.

The statistical analysis showed that FC was positively associated with UB. This means that patients are more likely to use Patient Online when they feel that they have the resources and knowledge enough to use it. This effect of FC was supported by several studies in the context of ePHRs [43,44,89]. In our study, this relationship was stronger for older patients with lower level of education and income and without internet access. In other words, these groups of people tend to place more importance on the presence of sufficient support and resources to use Patient Online. Although the moderating effect of age was supported in 1 CHITs study [76], this is the first study to investigate the moderating effects of education, income, and internet access in the context of ePHRs.

This study showed that BI positively associated with UB, that is, patients are more likely to use Patient Online when they intend to use it. This finding is consistent with findings of several studies in the context of ePHRs and CHITs [22,80,90-93].

Overall, the model accounted for 48% of the variance in UB. This moderate predictive power of the model indicates that there are other factors yet to be identified, which would account for the unexplained variance. Although the predictive power of the proposed model is comparable with the predictive power of the original UTAUT model (48%), it is higher than the predictive power of models proposed by other studies in the context of ePHRs: Hsieh [90] (42.7%) and Tavares and Oliveira [80] (26.8%).

### Theoretical and Practical Contributions

This is the first theory-based study to examine factors associated with patients’ use of ePHRs in England. Very few studies have utilized theories or models to understand the factors that impact patients’ use of ePHRs [21,24-27]. Furthermore, UTAUT was not employed in those few studies. Accordingly, this study contributes to the ePHR literature by adopting and testing UTAUT in the context of ePHRs, which can be used by the future studies in the context of ePHRs and CHITs.

This research and a study conducted by Tavares and Oliveira [80] are the only studies in the area of ePHRs that included both BI and UB in 1 model, and this is the best practice to study technology adoption [20,25]. Furthermore, our study contributes to the existing ePHR literature by being the first theory-based study to measure the UB objectively. In addition, this study is one of the few theory-based studies in the context of ePHRs that endeavored to minimize the common method bias by ensuring a gap in time between the main dependent variable (ie, use of Patient Online) and other variables. This research is the first study to shed light on the important role of moderators and mediators that explain the use of ePHRs, and this extends our understanding of factors that affect the adoption [27].

With respect to practical contributions, we have identified that PE and EE play a crucial role in forming patients’ intention to use Patient Online. Accordingly, developers should involve patients in the process of designing the system to consider functions and features that fit patients’ preferences and skills. Developers should pilot test the system with potential users before implementation [80,94]. As PPS is an influential predictor, developers should keep patient records as private as possible by protecting the platforms using security measures, such as strong firewalls, complex and long passwords, regular security reviews, and regular website updates.

To ensure that patients perceive the system as useful, easy to use, and secure, marketers should conduct promotional campaigns about functions and features of the system, its advantages, its ease of use, availability of different sources to support the use of the system, the security measures, the laws and regulations protecting patient privacy, and how patients can use it safely. As face-to-face communication may be one of the most effective channels in marketing to persuade potential adopters to adopt an innovation [95-97], physicians, nurses, and receptionists can play an important role in improving the publicity of Patient Online by informing patients about it in their communications. Marketers should focus more on younger males when conducting promotional campaigns regarding the benefits of the system, whereas they should concentrate more on older and less educated patients without internet access when initiating advertising campaigns regarding the ease of use of the system.

Patients who believed that organizational and technical infrastructure existed to support the use of Patient Online were more likely to use it. Therefore, to raise awareness of the infrastructure available, GPs could provide patients with manuals, Web-based assistance, technical support, and practical training sessions. This strategy is likely to be most effective with older patients and those with lower level of education and income.

Allowing patients to try a beta version of ePHRs could create a positive personal experience that may enhance their perceptions of usefulness and the ease of use of the system [26,75,78]. Thus, GPs should assist patients in using a beta version of Patient Online through a computer in a waiting room.

### Research Limitations

This study has limitations that need to be considered. Data were collected from 4 GPs, all implementing the same ePHRs (ie, SystemOnline); therefore, the findings of this study may not be applicable to other ePHRs (ie, Patient Access and Patient Services). However, the findings may still be generalizable to other systems because all the systems mentioned provide the same services to the patients, and all participants had not used any of them before. Therefore, they would be unlikely to have different perceptions about the different systems.

This study focused on assessing factors that affect patients’ initial use of ePHRs; therefore, the findings are not generalizable to the context of continuing use. This research focuses on initial use of ePHRs because Patient Online is still a new system in England and has a low adoption rate; therefore, it is better to focus on the initial use in this period. Furthermore, as this study is cross-sectional, the associations identified with patients’ initial use of ePHRs do not imply causality, and so further longitudinal research is required.

This research is subject to a sampling bias because of using convenience sampling technique to recruit the participants [36,98]. This study found that there was no statistically significant difference between the participants and the nonparticipants in terms of age, sex, and ethnicity. Consequently, it can be said that the findings are generalizable to practices similar to the 4 practices in this study.

It might have been appropriate to control for the effects from practices within the SEM. However, we found no differences in the demographics between the practices, indicating no evidence for practice level clustering. As these individual level factors are already included in the SEM, including clustering terms could lead to potential over adjustment.

### Recommendations for Future Research

Further studies are required to examine the applicability of the adapted model to other contexts. For example, research could investigate the applicability of the model to other providers of Patient Online (eg, Patient Access), specific platform (eg, mobiles, tablets, and computers), other settings (eg, hospitals), and other cities or countries.

Determining the factors that may influence the continuing use is important because long-term viability and eventual success of information technology depend on continued use [32,99-101]. Therefore, further primary studies and systematic reviews should be carried out to assess factors that affect the continuing use of ePHRs.

Further research is needed to explain the nonsignificant effect of SI demonstrated in this study. Previous studies demonstrated that the effect of SI depended on the type of processes of SI that people considered in their decisions (internalization, identification, and compliance) [37,84,102]. Thus, researchers may consider these 3 types of processes when assessing the effect of SI. Furthermore, researchers should develop new measures to assess the actual SI, such as the number of times a patient has been informed about the system by doctors, receptionists, friends, leaflets, posters, videos, and/or automated messages.

Although this study examined the effect of 4 moderators on most of the direct relationships, it did not examine their effects on the 2 indirect relationships (ie, EE→PE→BI and PPS→PE→BI). The effect of moderators on indirect relationships is called moderated mediation or conditional indirect effect [103-105]. To the best of our knowledge, the moderated mediating effect has not been examined in the context of ePHRs or CHITs. For this reason, future studies are required to test such an effect.

Finally, to increase the predictive power of the proposed model, future studies should consider adding other factors to the proposed model, such as patients’ satisfaction, patient activation level, health status, perceived severity, perceived susceptibility, awareness of Patient Online, compatibility, and results demonstrability.

### Conclusions

This study examined the main factors that affected patients’ use of ePHRs in England. The proposed model accounted for 48% of the variance in UB, indicating the existence of other, as yet unidentified, factors that influence adoption of ePHRs. Future studies should confirm the effect of the factors included in this model and identify additional factors. This study suggests that adoption rates are affected by key factors that should be taken into account for the successful implementation of ePHRs. For example, developers of ePHRs should involve patients in the process of designing the system to consider functions and features that fit patients’ preferences and skills, thereby, create a useful and easy to use system.

## Appendix

Multimedia Appendix 1 Selection of theory.

Multimedia Appendix 2 Conceptual definitions of constructs.

Multimedia Appendix 3 Theoretical foundation.

Multimedia Appendix 4 Characteristics of the GP practices.

Multimedia Appendix 5 Measures of constructs.

Multimedia Appendix 6 Questionnaire.

Multimedia Appendix 7 Results of construct reliability.

Multimedia Appendix 8 Results of convergent validity.

Multimedia Appendix 9 Intercorrelation coefficients and squared roots of AVE.

Multimedia Appendix 10 Item loadings and cross-loadings.

## Abbreviations

AVE: average variance extracted
BI: behavioral intention
CHIT: consumer health information technology
EE: effort expectancy
ePHR: electronic personal health record
FC: facilitating conditions
GFI: goodness-of-fit index
GP: general practice
PE: performance expectancy
PPS: perceived privacy and security
RMSEA: root mean square error of approximation
SEM: structural equation modeling
SI: social influence
SRMR: standardized root mean square residual
UB: use behavior
UTAUT: unified theory of acceptance and use of technology
