# Supplementary material for: Factors Affecting Patients’ Use of Electronic Personal Health Records in England: Cross-Sectional Study
Source: J Med Internet Res. 2019 Jul 31;21(7):e12373. doi: 10.2196/12373 (PMC6693305; doi:10.2196/12373)
Supplement: Multimedia Appendix 4 [file jmir_v21i7e12373_app4.docx]

| Features | Groups | Practice 1 | Practice 2 | Practice3 | Practice 4 |
| --- | --- | --- | --- | --- | --- |
| **Registered patients^a^** |  |  |  |  |  |
|  | - | 6193 | 6552 | 9235 | 9762 |
| **Online services^b^** |  |  |  |  |  |
|  | - | All services | All services | All services | All services |
| **Adoption rate** |  |  |  |  |  |
|  | - | 15% | 22% | 11% | 10% |
| **Location** |  |  |  |  |  |
|  | - | Bradford | Bradford | Bradford | Leeds |
| **Age** |  |  |  |  |  |
|  | 18-24 | 867 (14%) | 1068 (16.3%) | 1199 (13%) | 2716 (27.8%) |
|  | 25-34 | 1012 (16.3%) | 1323 (20.2%) | 1741 (19%) | 2955 (30.3%) |
|  | 35-44 | 883 (14.3%) | 1077 (16.4%) | 1694 (18.3%) | 1376 (14.1%) |
|  | 45-54 | 1045 (16.9%) | 1162 (17.7%) | 1530 (16.6%) | 919 (9.4%) |
|  | 55-64 | 940 (15%) | 922 (14.1%) | 1343 (14.5%) | 739 (7.6%) |
|  | 65-74 | 670 (11%) | 583 (8.9%) | 971 (10.5%) | 589 (6%) |
|  | 74+ | 776 (12.5%) | 417 (6.4%) | 757 (8.1%) | 468 (4.8%) |
| **Sex** |  |  |  |  |  |
|  | Male | 2962 (47.8%) | 3161 (48.2%) | 4607 (49.9%) | 5367 (55%) |
|  | Female | 3231 (52.2%) | 3391 (51.8) | 4628 (50.1%) | 4395 (45%) |
| **Ethnicity** |  |  |  |  |  |
|  | White | 85.3% | 77.7% | 78.2% | 76.2% |
|  | Asian | 7.3% | 17.6% | 16.3% | 10.9% |
|  | Black | 3.6% | 1.1% | 1.4% | 6.3% |
|  | Mixed | 3.4% | 2.6% | 2.7% | 4.4% |
|  | Others | 0.4% | 1% | 1.1% | 2.2% |
| ^a^All figures in the table represent patients who aged 18 years and older.  ^b^Online services include viewing records, booking appointments, ordering prescription. | | | | | |
