# Supplementary material for: Factors Affecting Patients’ Use of Electronic Personal Health Records in England: Cross-Sectional Study
Source: J Med Internet Res. 2019 Jul 31;21(7):e12373. doi: 10.2196/12373 (PMC6693305; doi:10.2196/12373)
Supplement: Multimedia Appendix 5 [file jmir_v21i7e12373_app5.docx]

| **Constructs** | **Measures/ Indicators** | | **Sources** |
| --- | --- | --- | --- |
| **Performance Expectancy (PE)** | PE1 | I think Patient Online will be useful in managing my health care. | Venkatesh et al. (2012) |
|  | PE2 | I think using Patient Online would help me do things (e.g. booking appointments and ordering repeat prescriptions) less quickly. | Venkatesh et al. (2012) |
|  | PE3 | I believe using Patient Online would enhance my effectiveness in managing my health care. | Venkatesh et al. (2012) |
| **Effort Expectancy**  **(EE)** | EE1 | I think learning how to use Patient Online would be difficult for me. | Venkatesh et al. (2012) |
|  | EE2 | I expect my interaction with Patient Online would be clear and understandable. | Venkatesh et al. (2012) |
|  | EE3 | I believe I would find Patient Online easy to use. | Venkatesh et al. (2012) |
|  | EE4 | I believe it would be difficult for me to become skilful at using Patient Online. | Venkatesh et al. (2012) |
| **Social Influences**  **(SI)** | SI1 | People who are important to me would think that I should use Patient Online. | Venkatesh et al. (2012) |
|  | SI2 | People whose opinions that I value would prefer that I use Patient Online. | Venkatesh et al. (2012) |
|  | SI3 | People who influence my behaviour would think that I should use Patient Online. | Venkatesh et al. (2012) |
| **Facilitating Conditions**  **(FC)** | FC1 | I think I have the resources necessary to use Patient Online. | Venkatesh et al. (2012) |
|  | FC2 | I think I have the knowledge necessary to use Patient Online. | Venkatesh et al. (2012) |
|  | FC3 | Patient Online is compatible with other web-based services I use (e.g. Amazon, eBay, or Internet banking). | Venkatesh et al. (2012) |
|  | FC4 | I can get help from others when I have difficulties using Patient Online. | Venkatesh et al. (2012) |
|  | FC5 | I believe there is always a helpdesk for help in solving problems with the Patient Online website. | Or (2008) |
| **Perceived Privacy & Security**  **(PPS)** | PPS1 | I would feel that Patient Online is vulnerable. | Whetstone & Goldsmith (2009) |
|  | PPS2 | I would feel that health information maintained in my Patient Online would be protected. | Whetstone & Goldsmith (2009) |
|  | PPS3 | I would feel that my health record will be kept private. | Whetstone & Goldsmith (2009) |
|  | PPS4 | I am worried about privacy issues when using Patient Online. | Rao (2014) |
|  | PPS5 | I believe Patient Online can ensure my personal security if it is password protected. | Rao (2014) |
| **Behavioural Intention**  **(BI)** | BI1 | I intend to use Patient Online in the next 6 months. | Venkatesh et al. (2003) |
|  | BI2 | I predict I would use Patient Online in the next 6 months. | Venkatesh et al. (2003) |
|  | BI3 | I plan to use Patient Online in the next 6 months. | Venkatesh et al. (2003) |
| **Age** | Ag | What is your age? | Richards (2012) |
| **Sex** | Sx | What is your sex? | Richards (2012) |
| **Ethnicity** | Ethn | What is your ethnicity? | Noblin (2010) |
| **Income** | Inc | What is your household income level (£/year)? | Richards (2012) |
| **Education** | Edu | What is the highest level of education that you have completed? | Rao (2014) |
| **Internet Access** | Int | Do you have internet access where you live? | Logue (2011) |
