# Supplementary material for: Factors Affecting Patients’ Use of Electronic Personal Health Records in England: Cross-Sectional Study
Source: J Med Internet Res. 2019 Jul 31;21(7):e12373. doi: 10.2196/12373 (PMC6693305; doi:10.2196/12373)
Supplement: Multimedia Appendix 6 [file jmir_v21i7e12373_app6.docx]

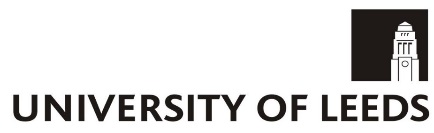


General practice: _ _ _ _ _ _ _ _ _ _ _ _ _ _

Participant Identification Number: _ _ _ _ _

**Questionnaire**

**Introduction**

**What is Patient Online?**

It is an NHS England programme designed to support GP practices to offer and promote online services to patients, including access to some information in their medical records, booking appointments and ordering repeat prescriptions.

**Note:** Before starting the survey, you should note that some questions may look similar. So, please read them carefully and answer all the questions. Also, to keep it confidential, please do not write your name on any part of this questionnaire.

**Part 1: Please indicate the extent to which you agree with the following statements for each item, using the scale below:**

**1: Strongly disagree 2: Disagree 3: Slightly disagree 4: Neutral**

**5: Slightly agree 6: Agree 7: Strongly agree**

|  |  | **Strongly Disagree** | **Disagree** | **Slightly Disagree** | **Neutral** | **Slightly** **Agree** | **Agree** | **Strongly** **Agree** |
| --- | --- | --- | --- | --- | --- | --- | --- | --- |
| 1. | I intend to use Patient Online in the next 6 months. | 1 | 2 | 3 | 4 | 5 | 6 | 7 |
| 2. | I believe I would find Patient Online easy to use. | 1 | 2 | 3 | 4 | 5 | 6 | 7 |
| 3. | I would feel that Patient Online is vulnerable. | 1 | 2 | 3 | 4 | 5 | 6 | 7 |
| 4. | I plan to use Patient Online in the next 6 months. | 1 | 2 | 3 | 4 | 5 | 6 | 7 |
| 5. | I am worried about privacy issues when using Patient Online. | 1 | 2 | 3 | 4 | 5 | 6 | 7 |
| 6. | I think I have the knowledge necessary to use Patient Online. | 1 | 2 | 3 | 4 | 5 | 6 | 7 |
| 7. | I predict I would use Patient Online in the next 6 months. | 1 | 2 | 3 | 4 | 5 | 6 | 7 |
| 8. | People who are important to me would think that I should use Patient Online. | 1 | 2 | 3 | 4 | 5 | 6 | 7 |
| 9. | I can get help from others when I have difficulties using Patient Online. | 1 | 2 | 3 | 4 | 5 | 6 | 7 |
| 10. | I think learning how to use Patient Online would be difficult for me. | 1 | 2 | 3 | 4 | 5 | 6 | 7 |
| 11. | I would feel that my health record will be kept private. | 1 | 2 | 3 | 4 | 5 | 6 | 7 |
| 12. | I believe there is always a helpdesk for help in solving problems with the Patient Online website. | 1 | 2 | 3 | 4 | 5 | 6 | 7 |
| 13. | Patient Online is compatible with other web-based services I use (e.g. Amazon, eBay, or Internet banking). | 1 | 2 | 3 | 4 | 5 | 6 | 7 |
| 14. | I believe it would be difficult for me to become skilful at using Patient Online. | 1 | 2 | 3 | 4 | 5 | 6 | 7 |
| 15. | People who influence my behaviour would think that I should use Patient Online. | 1 | 2 | 3 | 4 | 5 | 6 | 7 |
| 16. | I think I have the resources necessary to use Patient Online (e.g. internet, computer, IPad). | 1 | 2 | 3 | 4 | 5 | 6 | 7 |
| 17. | I would feel that health information maintained in my Patient Online would be protected. | 1 | 2 | 3 | 4 | 5 | 6 | 7 |
| 18. | I think using Patient Online would help me do things (e.g. booking appointments and ordering repeat prescriptions) less quickly. | 1 | 2 | 3 | 4 | 5 | 6 | 7 |
| 19 | I think Patient Online will be useful in managing my health care. | 1 | 2 | 3 | 4 | 5 | 6 | 7 |
| 20 | I believe Patient Online can ensure my personal security if it is password protected. | 1 | 2 | 3 | 4 | 5 | 6 | 7 |
| 21. | I expect my interaction with Patient Online would be clear and understandable. | 1 | 2 | 3 | 4 | 5 | 6 | 7 |
| 22. | People whose opinions that I value would prefer that I use Patient Online. | 1 | 2 | 3 | 4 | 5 | 6 | 7 |
| 23. | I believe using Patient Online would enhance my effectiveness in managing my health care. | 1 | 2 | 3 | 4 | 5 | 6 | 7 |

**If you have any comments to help us understand more about why people may or may not use Patient Online, please would you add them here?**

**Part 2: Personal Information (Please tick the relevant box; one tick on each question).**

1. **What is your sex?**

Male Female

1. **What is your age?** _______
2. **What is the highest level of education that you have completed?**

Up to secondary school Secondary school College Degree

Bachelor Degree Master Degree Doctoral Degree

1. **What is your ethnicity?**

White Asian or Asian British Black or Black British

Mixed or Multiple Others

1. **Do you have internet access where you live?**

Yes No

1. **What is your household income level (£/year)?**

Less than 20,000 20,000-29,999 30,000-39,999

40,000-49,999 50,000-59,999 60,000 or more

Prefer not to say

**Thank you for your time to complete this questionnaire.**

**If you have any comments regarding the survey do not hesitate to contact me on the following contact details:**

**Phone: 0113 343 0896**

**Email:** [**umaaea@leeds.ac.uk**](mailto:umaaea@leeds.ac.uk)
