# Supplementary material for: Factors Affecting Patients’ Use of Electronic Personal Health Records in England: Cross-Sectional Study
Source: J Med Internet Res. 2019 Jul 31;21(7):e12373. doi: 10.2196/12373 (PMC6693305; doi:10.2196/12373)
Supplement: Multimedia Appendix 7 [file jmir_v21i7e12373_app7.docx]

| Constructs | Cronbach’s alpha (α)^a^ | Composite Reliability (CR)^a^ | Average Variance Extracted (AVE)^b^ |
| --- | --- | --- | --- |
| **Performance expectancy** |  |  |  |
|  | 0.962 | 0.962 | 0.895 |
| **Effort expectancy** |  |  |  |
|  | 0.961 | 0.962 | 0.863 |
| **Social influence** |  |  |  |
|  | 0.946 | 0.948 | 0.858 |
| **Facilitating conditions** |  |  |  |
|  | 0.940 | 0.942 | 0.843 |
| **Perceived privacy and security** |  |  |  |
|  | 0.941 | 0.942 | 0.845 |
| **Behavioural intention** |  |  |  |
|  | 0.962 | 0.963 | 0.898 |
| ^a^Cut-off point of ≥0.70  ^b^Cut-off point of ≥0.50 | | | |
