# Supplementary material for: Factors Affecting Patients’ Use of Electronic Personal Health Records in England: Cross-Sectional Study
Source: J Med Internet Res. 2019 Jul 31;21(7):e12373. doi: 10.2196/12373 (PMC6693305; doi:10.2196/12373)
Supplement: Multimedia Appendix 8 [file jmir_v21i7e12373_app8.docx]

| Latent Constructs | Items | Factor loading^a^ | AVE^b^ |
| --- | --- | --- | --- |
|  |  |  |  |
| **Performance Expectancy** |  |  |  |
|  | PE1 | 0.97 | 0.895 |
|  | PE2 | 0.95 |  |
|  | PE3 | 0.92 |  |
| **Effort Expectancy** |  |  |  |
|  | EE1 | 0.95 | 0.863 |
|  | EE2 | 0.92 |  |
|  | EE3 | 0.95 |  |
|  | EE4 | 0.90 |  |
| **Social Influences** |  |  |  |
|  | SI1 | 0.94 | 0.858 |
|  | SI2 | 0.96 |  |
|  | SI3 | 0.88 |  |
| **Facilitating Conditions** |  |  |  |
|  | FC1 | 0.97 | 0.843 |
|  | FC2 | 0.93 |  |
|  | FC3 | 0.86 |  |
| **Perceived Privacy & Security** |  |  |  |
|  | PPS1 | 0.95 | 0.845 |
|  | PPS2 | 0.94 |  |
|  | PPS3 | 0.86 |  |
| **Behavioural Intention** |  |  |  |
|  | BI1 | 0.97 | 0.898 |
|  | BI2 | 0.95 |  |
|  | BI3 | 0.91 |  |
| ^a^Cut-off point of ≥0.70  ^b^Cut-off point of ≥0.50 | | | |
