# Supplementary material for: Factors Affecting Patients’ Use of Electronic Personal Health Records in England: Cross-Sectional Study
Source: J Med Internet Res. 2019 Jul 31;21(7):e12373. doi: 10.2196/12373 (PMC6693305; doi:10.2196/12373)
Supplement: Multimedia Appendix 9 [file jmir_v21i7e12373_app9.docx]

| **Latent constructs** | **PE** | **EE** | **SI** | **FC** | **PPS** | **BI** |
| --- | --- | --- | --- | --- | --- | --- |
| **PE** | **0.946** |  |  |  |  |  |
| **EE** | 0.454 | **0.929** |  |  |  |  |
| **SI** | 0.647 | 0.501 | **0.926** |  |  |  |
| **FC** | 0.563 | 0.837 | 0.530 | **0.918** |  |  |
| **PPS** | 0.538 | 0.525 | 0.701 | 0.541 | **0.919** |  |
| **BI** | 0.493 | 0.506 | 0.484 | 0.573 | 0.621 | **0.947** |
| ^a^Off-diagonal values are the estimates of inter-correlation between the latent constructs (Cut-off point of <0.85).  ^b^Diagonal values are squared roots of AVE. | | | | | | |
